# Comparative Evaluation of Setup Errors Using Planar Imaging and Cone Beam Computed Tomography: Feasibility of Asymmetric Planning Margins

**DOI:** 10.7759/cureus.88599

**Published:** 2025-07-23

**Authors:** Eleni Styliani Katrakylidou, Emmanouil Papanastasiou, Georgios Plataniotis, Pericles Bousbouras, Alexandra Kriari, Ioannis Tzitzikas, Panagiotis D Bamidis, Anastasios Siountas

**Affiliations:** 1 Department of Medical Physics and Digital Innovation, American Hellenic Educational Progressive Association (AHEPA) University Hospital, Thessaloniki, GRC; 2 Department of Medical Physics and Digital Innovation, School of Medicine, Faculty of Health Sciences, Aristotle University of Thessaloniki, Thessaloniki, GRC; 3 Department of Radiation Oncology, American Hellenic Educational Progressive Association (AHEPA) University Hospital, Thessaloniki, GRC; 4 Department of Radiation Oncology, School of Medicine, Faculty of Health Sciences, Aristotle University of Thessaloniki, Thessaloniki, GRC; 5 Laboratory of Medical Physics and Digital Innovation, School of Medicine, Faculty of Health Sciences, Aristotle University of Thessaloniki, Thessaloniki, GRC

**Keywords:** asymmetric margins, cbct imaging, planar kv imaging, setup errors, van herk

## Abstract

Background

Accurate patient positioning in radiotherapy is critical to ensure adequate dose delivery to tumors and minimize radiation dose to normal tissues. Two image-guided radiotherapy (IGRT) techniques, planar and cone beam computed tomography (CBCT), are commonly used to measure patient setup deviations.

Objective

This study aims to compare the mean setup errors between planar and CBCT procedures in various irradiated areas, across the three orthogonal axes and at different times of day (morning-afternoon), and to evaluate whether the use of asymmetric planning margins between CTV and PTV is statistically justified.

Methods

A total of 178 patients, irradiated in four anatomical sites, were enrolled in the study. They were irradiated for prostate (n = 51, 28.6%), head and neck (n = 48, 27.0%), lung (n = 45, 25.3%), and metastatic brain tumors (n = 34, 19.1%). Each patient underwent both planar and CBCT imaging for the first three treatment days and every five days thereafter. Bone anatomy matching was carried out with both imaging techniques. Paired-samples t-tests, independent samples t-tests, and multivariate analysis of variance (MANOVA) were used. The van Herk formula was employed to determine symmetric and asymmetric planning margins based on error distributions. Statistical significance was defined at α = 0.05 (5%).

Results

CBCT demonstrated statistically higher deviations than planar imaging in specific X-Y-Z axes and anatomical sites, namely, along the X-axis in the prostate, lung, and cranial regions and along Z-axis in the head and neck, lung, and cranial regions. Time of treatment did not affect our results. Statistically significant differences between positive and negative errors in some regions might support the use of asymmetric van Herk margins.

Conclusion

According to our study, CBCT is more sensitive in detecting setup deviations/errors compared to planar imaging in certain anatomical regions and dimensions. Asymmetric planning margins may increase treatment precision and reduce the irradiated volume of normal tissues, particularly in lung and cranial irradiation.

## Introduction

Image-guided radiotherapy (IGRT) has become an integral component in modern radiotherapy (RT), enabling precise targeting of tumor volumes while sparing healthy tissues. Two widely used imaging modalities for IGRT include two-dimensional planar kilovoltage (kV) imaging and three-dimensional cone beam computed tomography (CBCT). Each modality offers distinct advantages and limitations in terms of image quality, setup verification, and time efficiency [1-5].

Planar kV imaging, although fast and widely accessible, provides limited volumetric information. Conversely, CBCT offers three-dimensional anatomical visualization, which enhances soft tissue contrast and target localization accuracy [1-5]. However, CBCT may introduce higher variability in patient setup due to factors such as motion artifacts and extended imaging duration [1,3-6]. These differences raise the question of whether CBCT's theoretical advantages translate into clinically meaningful improvements in setup precision [1,5,7].

Several studies have compared CBCT and planar imaging across various anatomical sites, such as the prostate, lung, and head and neck, revealing variable outcomes in terms of absolute mean setup errors. Yet few studies provide a unified statistical analysis that considers all three axes (X, Y, Z), anatomical areas, and time-of-day variations within the same cohort of patients [5,8,9].

Another critical component in RT planning involves the estimation of CTV-PTV margins. The conventional van Herk formula provides symmetric margins to account for systematic and random setup errors [10-12]. Recent reports suggested asymmetric margins to improve conformity and reduce exposure of healthy tissue, especially in cases where directional setup error distributions are non-uniform [6,13-15].

The present study aims to compare the absolute mean setup errors derived from CBCT and planar imaging across four anatomical areas (prostate, head and neck, lung, and cranial), considering three spatial dimensions (X, Y, Z) and temporal factors (day, morning vs. afternoon). Time-of-day analysis was included to assess potential fatigue-related or workflow-associated variations in setup accuracy.

In summary, the primary objectives of this study are (1) to compare mean setup errors between planar kV and CBCT imaging across anatomical sites, spatial axes, and time of day and (2) to evaluate whether statistically significant directional errors justify the implementation of asymmetric van Herk planning margins in clinical practice.

## Materials and methods

A total of 178 (51 prostate (28.6%), 48 head and neck (27.0%), 45 lung (25.3%), and 34 cranial (19.1%)) irradiation patients receiving RT were included in the analysis. According to our departmental protocol, each patient underwent both CBCT and planar kV imaging for the first three treatment days and every five days thereafter. The total number of images acquired for each patient varied according to the total number of fractions of the particular RT schedule.

For CBCT images, bone anatomy matching was performed automatically with the vendor-supplied registration software. In the case of planar kV imaging, matching was conducted manually by senior radiographers and reviewed by radiation oncologists prior to correction; these “corrections” were the data collected for the present study.

Standard immobilization tools such as thermoplastic mask for head and neck and cranial RT and commercial thorax positional systems for chest RT were used. Imaging acquisition protocols (e.g., field of view (FOV), exposure settings) and immobilization methods were standardized to the greatest extent possible across anatomical areas. For CBCT imaging, the acquisition protocol included a tube voltage of 120 kVp, tube current of 16 mA, exposure time of 16 ms per frame, and a total of 183 projections over a 200-degree gantry rotation for a standard FOV of 27 cm. The slice thickness was set to 2 mm for all scans. For planar kV imaging, exposure settings were adjusted according to anatomical site: typical settings included 70-85 kVp and 3.2-5 mAs for prostate and head and neck regions, and 100-120 kVp with 2.5-4 mAs for thoracic and cranial imaging. All exposures adhered to the clinical protocols and manufacturer recommendations, with additional adjustments as required for patient size and image quality. Detailed acquisition settings were kept consistent throughout the study, and further protocol deviations were documented. 

The study evaluated absolute mean setup errors across three orthogonal axes (X, Y, Z) and considered additional stratifications by anatomical area and time of treatment (to identify any possible differences between daily shifts, morning or afternoon). For each imaging modality and anatomical region, the setup error was calculated as the deviation between planned and actual patient position.

All data were statistically analyzed using IBM SPSS Version 27 (IBM Corp., Armonk, NY). Parametric tests were applied, as the central limit theorem ensures approximation of normality for the sample sizes used. In the first step, paired-samples t-tests were performed to compare mean setup errors between CBCT and planar imaging across each spatial axis, anatomical site, and time of treatment.

Having examined the presence of significant differences between the two imaging methods in the absolute mean setup errors as well as the effect of treatment time, a mixed analysis of Variance (MANOVA) was further conducted to assess possible differences among mean setup errors by anatomical area, time and day, accounting also for interaction effects among the independent factors (area, time) and the dependent factor (day). Mauchly’s test was used to examine the sphericity assumption, and adjustments were made where necessary.

To explore the feasibility of asymmetric planning margins, the individual mean setup errors were further split into positive (anterior, right, superior) and negative (posterior, left, inferior). The presence of statistically significant differences between these subgroups was evaluated using independent samples t-tests. The null hypothesis in Levene’s test assumes statistical equality of variances across the comparison groups. Depending on the outcome of this test, the appropriate version of the independent samples t-test is selected accordingly. Where significant, asymmetric van Herk margins were calculated separately for positive and negative directions.

As it is well known, van Herk suggested the following formula:

 $2.5 \Sigma + 0.7 \sigma$

where Σ corresponds to the systematic error of the population and σ to the random errors of the population. Clinical target volume (CTV)-planning target volume (PTV) margins are calculated to allow that 90% of patients receive 95% of the prescribed dose at CTV, resulting in 1% TCP loss in the population.

The systematic error of the population corresponds to the standard deviation of the individual means, and the random error of the population is the mean of the individual standard deviations.

All statistical analyses were conducted at a 5% significance level (α = 0.05).

## Results

**Figure 1:**
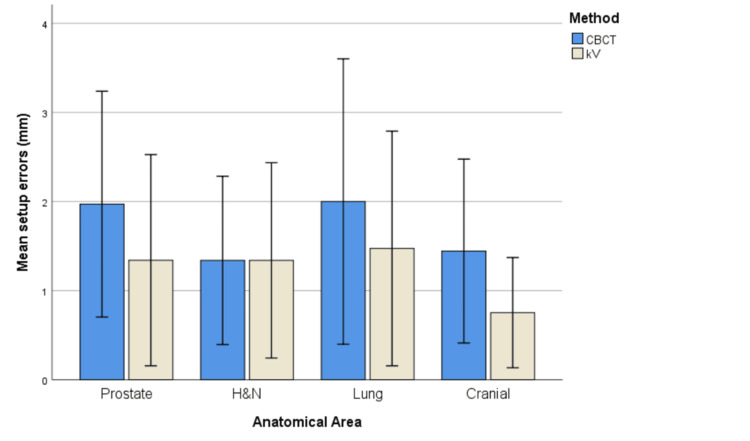
Mean absolute setup errors per anatomical area for X axis Error bars represent ±1 standard deviation (SD).

**Figure 2:**
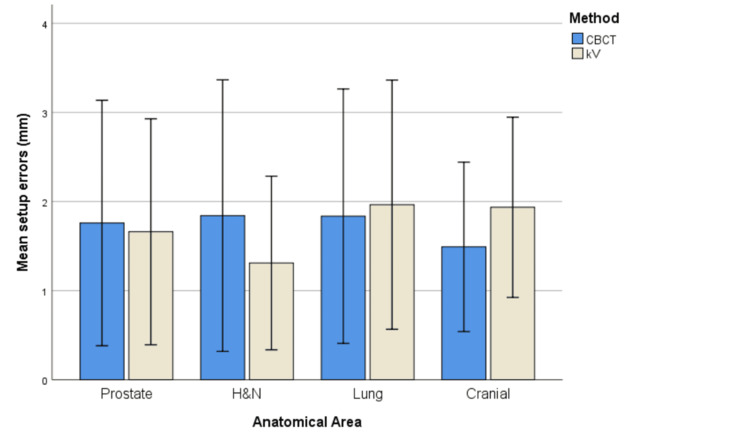
Mean absolute setup errors per anatomical area for Y axis Error bars represent ±1 standard deviation (SD).

**Figure 3:**
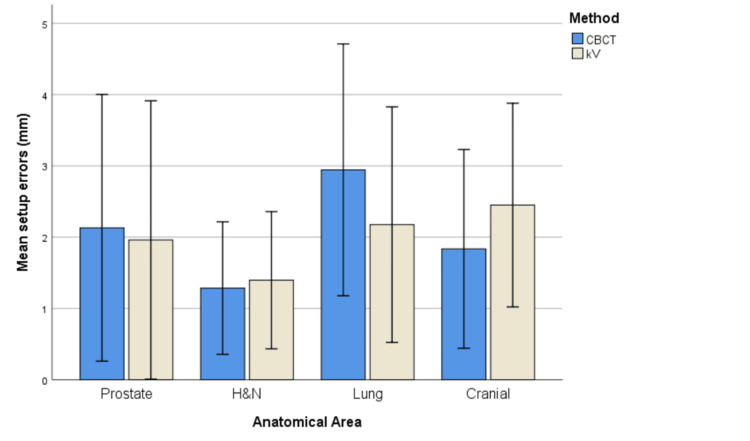
Mean absolute setup errors per anatomical area for Z axis Error bars represent ±1 standard deviation (SD).

The bar charts below attempt to visualize the differences in the mean absolute setup errors between the two imaging methods per axis and anatomical area (Figures 1-3).

It is obvious that for the X axis, CBCT generally seems to produce higher mean absolute setup errors than kV for all anatomical areas. However, regarding the other two axes, the pattern is rather mixed depending on the anatomical area.

Initially, a paired-samples t-test was applied to investigate statistically significant differences between the CBCT and kV imaging methods for all anatomical areas and time of treatment. The results are reported in Table 1 below.

**Table 1:** Paired-samples t-test results for differences between mean absolute setup errors between image-guided procedures CBCT: cone beam computed tomography; kV: kilovoltage imaging procedure; mm: millimeters; t: t-statistic values Results were considered statistically significant if p < 0.05 and are in bold.

Anatomical area	Axis	Treatment time	Mean difference CBCT - kV (mm)	t	p
Prostate	X	Morning	0.81	4.225	<0.001
		Afternoon	0.88	8.887	<0.001
		Total	0.84	6.697	<0.001
	Y	Morning	0.01	0.039	0.969
		Afternoon	0.04	0.234	0.818
		Total	0.02	0.121	0.904
	Z	Morning	-0.54	-2.065	0.047
		Afternoon	-0.04	-0.171	0.866
		Total	-0.36	-1.871	0.067
Head and neck	X	Morning	0.56	2.516	0.018
		Afternoon	0.59	1.402	0.179
		Total	0.58	2.750	0.008
	Y	Morning	-0.41	-1.182	0.247
		Afternoon	-1.02	-3.058	0.007
		Total	-0.64	-2.542	0.014
	Z	Morning	-1.01	-4.885	<0.001
		Afternoon	-0.66	-2.343	0.032
		Total	-0.88	-5.267	<0.001
Lung	X	Morning	1.17	5.661	<0.001
		Afternoon	1.22	3.914	0.002
		Total	1.18	6.961	<0.001
	Y	Morning	0.21	0.846	0.404
		Afternoon	0.13	0.417	0.684
		Total	0.18	0.950	0.347
	Z	Morning	0.66	2.609	0.014
		Afternoon	0.36	1.394	0.187
		Total	0.57	2.962	0.005
Cranial	X	Morning	0.90	4.476	<0.001
		Afternoon	1.34	2.647	0.023
		Total	1.06	4.808	<0.001
	Y	Morning	0.57	3.811	<0.001
		Afternoon	0.42	1.384	0.194
		Total	0.52	3.643	<0.001
	Z	Morning	-0.55	-4.000	<0.001
		Afternoon	-0.67	-1.821	0.096
		Total	-0.59	-3.845	<0.001

In the next step of the analysis, the MANOVA was applied to examine potential differences in mean setup errors by anatomical area, time, and day, while also accounting for interaction effects among the independent factors (anatomical area, time) and the dependent factor (day). Mauchly’s test rejected the null hypothesis of equal variances among all combinations of related groups (sphericity). Hence, the Greenhouse-Geisser adjustment was applied to reduce the risk of type I error. The results are presented in Table 2 below.

**Table 2:** Multivariate analysis of variance (MANOVA) results for potential differences in mean setup errors by anatomical area, time, and day CBCT: cone beam computed tomography; F: F-statistic values; kV: kilovoltage imaging procedure Results were considered statistically significant if p < 0.05 and are in bold.

Spatial dimension	Method	Anatomical area	Time	Day	Day * area	Area * time	Day * time	Day * time * area
		F (p)	F (p)	F (p)	F (p)	F (p)	F (p)	F (p)
Χ	CBCT	4.658 (0.004)	0.754 (0.387)	0.614 (0.683)	1.235 (0.242)	1.694 (0.170)	0.410 (0.835)	0.887 (0.576)
	kV	2.185 (0.092)	0.437 (0.509)	0.551 (0.735)	1.274 (0.213)	1.555 (0.202)	0.513 (0.764)	1.521 (0.092)
Υ	CBCT	1.003 (0.393)	2.182 (0.141)	5.276 (0.000)	1.091 (0.362)	1.903 (0.131)	0.617 (0.671)	1.227 (0.252)
	kV	5.150 (0.002)	0.630 (0.429)	3.530 (0.005)	0.766 (0.706)	1.615 (0.188)	0.611 (0.679)	1.230 (0.248)
Ζ	CBCT	14.867 (0.000)	1.113 (0.293)	12.038 (0.000)	0.907 (0.534)	0.674 (0.569)	1.210 (0.306)	0.393 (0.960)
	kV	8.606 (0.000)	1.712 (0.193)	15.508 (0.000)	1.674 (0.058)	0.753 (0.533)	1.860 (0.117)	0.952 (0.497)

To improve the accuracy of planning margin estimation and build upon prior findings, this study investigates potential statistically significant differences between positive and negative mean setup errors. Table 3 presents the results from the comparison between positive and negative mean setup errors.

**Table 3:** t-test for independent samples: comparison of positive and negative mean setup errors CBCT: cone beam computed tomography; kV: kilovoltage imaging procedure; mm: millimeters; t: t-statistic values. Results were considered statistically significant if p < 0.05 and are in bold.

Anatomical area	Procedure	Axis	Mean setup errors (mm)		Mean difference (mm)	t	p
			Positive (+)	Negative (-)			
Prostate	CBCT	X	2.04	1.60	0.44	0.899	0.373
		Y	1.62	1.82	-0.20	-0.487	0.628
		Z	2.43	1.71	0.72	1.359	0.180
	kV	X	1.36	1.29	0.07	0.177	0.860
		Y	1.76	1.63	0.13	0.329	0.744
		Z	2.09	1.61	0.48	0.793	0.432
Head and neck	CBCT	X	1.35	1.32	0.03	0.073	0.942
		Y	1.59	1.92	-0.33	-0.652	0.518
		Z	1.22	1.39	-0.17	-0.624	0.536
	kV	X	1.28	1.41	-0.13	-0.401	0.691
		Y	1.03	1.53	-0.50	-1.810	0.077
		Z	1.55	0.80	0.75	3.492	<0.001
Lung	CBCT	X	2.34	1.06	1.28	3.651	<0.001
		Y	0.82	2.16	-1.34	-2.948	0.005
		Z	3.19	1.37	1.82	2.484	0.017
	kV	X	1.80	1.14	0.66	1.736	0.091
		Y	0.87	2.24	-1.37	-2.835	0.007
		Z	2.41	0.62	1.79	2.647	0.011
Cranial	CBCT	X	1.74	0.62	1.12	4.215	<0.001
		Y	0.43	1.82	-1.39	-7.156	<0.001
		Z	-	-	-	-	-
	kV	X	0.81	0.69	0.12	0.549	0.587
		Y	0.73	2.05	-1.32	-2.290	0.029
		Z	-	-	-	-	-

Table 4 illustrates the symmetric planning margins according to the van Herk method as well as the new asymmetric margins, based on the aforementioned statistically significant differences.

**Table 4:** Symmetric and asymmetric van Herk planning margins CBCT: cone beam computed tomography; kV: kilovoltage imaging procedure; mm: millimeters

Anatomical area	Procedure	Spatial dimension	Symmetric planning margins (mm)	Asymmetric planning margins (mm)	
				Positive	Negative
Prostate	CBCT	Χ	6.5	-	-
		Υ	7.5	-	-
		Ζ	9.5	-	-
	kV	Χ	6.1	-	-
		Υ	7.0	-	-
		Ζ	8.7	-	-
Head and neck	CBCT	Χ	5.8	-	-
		Υ	6.9	-	-
		Ζ	5.4	-	-
	kV	Χ	6.2	-	-
		Υ	5.6	-	-
		Ζ	4.6	3.8	2.5
Lung	CBCT	Χ	6.5	6.2	2.8
		Υ	6.0	4.0	5.6
		Ζ	7.3	6.5	5.4
	kV	Χ	6.2	-	-
		Υ	6.0	5.2	5.3
		Ζ	6.1	6.1	3.4
Cranial	CBCT	Χ	4.3	3.7	2.6
		Υ	4.1	2.5	3.5
		Ζ	4.4	-	-
	kV	Χ	3.4	-	-
		Υ	4.3	2.8	4.1
		Ζ	4.6	-	-

## Discussion

The present study provides a comprehensive analysis of setup errors observed in IGRT, comparing CBCT and planar kV imaging across multiple anatomical areas.

According to Table 1, statistically significant differences (p < 0.05) were observed between the two imaging methodologies in the prostate region, along the X-axis, both total and between the morning and afternoon time of treatment. The mean differences in these comparisons are positive, indicating that CBCT tends to produce greater deviations compared to kV imaging. Furthermore, in the same anatomical area, a statistically significant difference is also detected along the Z-axis in the morning irradiation, where the mean difference is negative, suggesting that along this dimension, CBCT tends to yield smaller deviations than kV.

In the head and neck area, statistically significant differences between the two methods were identified along the X-axis overall and during morning irradiation, along the Y-axis in the afternoon and overall, and along the Z-axis during the morning, afternoon, and overall. As regards the X dimension in particular, the positive mean (significant) differences observed suggest that CBCT results in greater deviations than kV. In contrast, along the Y dimension, the statistically significant comparisons yield negative mean differences, indicating that CBCT tends to produce smaller deviations compared to kV. Similarly, in the Z dimension, the negative mean differences in the statistically significant comparisons support the evidence that CBCT generates smaller deviations than kV.

In the lung area, statistically significant differences (p < 0.05) between the two methods were identified along the X-axis (morning, afternoon, and overall) and the Z-axis (morning and overall). In all instances, the mean differences were positive, indicating that CBCT consistently exhibited greater deviations than kV imaging in these directions.

Finally, in the cranial area, significant differences are observed between the two methods along the X-axis (morning, afternoon, and overall), the Y-axis (morning and overall), and the Z-axis (morning only). In both the X and Y dimensions, the mean differences are positive, indicating that CBCT measurements deviate more than those of kV. However, in the Z dimension, the significant negative differences reveal that CBCT provides smaller deviations compared to kV.

Summing up, the above-mentioned findings indicate that CBCT generally produces higher mean absolute setup errors than planar imaging in specific spatial dimensions, especially along the X-axis for prostate, lung, and cranial treatments [1-6,9,15]. These findings align with previous studies that have highlighted the superior soft-tissue visualization of CBCT at the cost of increased variability due to motion artefacts and acquisition time [1,3-5]. Notably, the head and neck area showed mixed results, with CBCT offering smaller deviations in the Y and Z axes, suggesting improved soft tissue matching in this area [3,7,16,17]. Despite the theoretical advantages of CBCT, the lack of significant differences in the Y-axis across most areas suggests that setup errors in this axis are less sensitive to imaging modality. This observation is consistent with earlier reports by Wang et al. and Yartsev et al., which demonstrated limited inter-method variability in lateral setup deviations [1,18].

The observed differences between CBCT and planar kV imaging can be attributed to several technical and procedural factors intrinsic to each modality. CBCT provides three-dimensional volumetric data with improved soft-tissue contrast, which can result in more accurate detection of setup errors but also introduces increased sensitivity to patient motion and potential artifacts due to longer acquisition times. In contrast, planar kV imaging acquires images more rapidly and is less susceptible to motion artifacts, but its two-dimensional nature limits the visualization of soft-tissue structures, potentially resulting in underestimation of certain deviations. Additionally, anatomical region-specific challenges, such as respiratory motion in lung cases or variable immobilization in cranial treatments, may affect each imaging technique differently, further contributing to the observed differences in setup error detection.

Useful indications were next obtained by means of MANOVA analysis. The results demonstrate that anatomical area is a key contributor to setup error variance, particularly in the X and Z dimensions, reinforcing the need for area-specific quality assurance protocols [7,8,19,20]. Additionally, the significant day-to-day variability in Y and Z dimensions underscores the value of daily image guidance, while the absence of statistically significant differences between morning and afternoon treatments confirms the consistency of imaging procedures throughout the day. This finding supports operational reliability in daily clinical practice throughout daily shifts [5,8,9,21]. More particularly, according to Table 2, the analysis suggests that the independent factor “area” has a statistically significant effect on the deviations along the X-axis for both imaging methods, along the Y-axis for kV, and along the Z-axis for both methods. In contrast, the independent factor “time of treatment” does not appear to significantly influence the size of setup errors in any dimension or method. The dependent factor “day of treatment” is found to exert a statistically significant effect on deviations along the Y and Z axes for both CBCT and kV methods. Finally, no statistically significant interaction effects are identified between the examined factors in any dimension for either method.

As is well known, the conventional "van Herk" method is typically employed in determining planning margins, defining the irradiated area with symmetric margins in all dimensions [10-12]. In this direction, a notable aspect of this study is the exploration of positive vs. negative setup errors. Statistically significant differences in directional deviations support the implementation of asymmetric van Herk margins in select cases [6,13,15]. This is particularly relevant for lung and cranial RT, where directional setup shifts may introduce systematic bias in treatment delivery. These findings are in line with the recent literature advocating for adaptive margin design to optimize dosimetric coverage and minimize normal tissue exposure [11,13,14].

More specifically, the results reported in Table 3 reveal no statistically significant differences between positive and negative mean set-up errors for the “prostate” area. In contrast, for the “head and neck” area, a significant difference was detected in the kV imaging procedure along the Z axis. Similarly, in the “lung” area, statistically significant differences were observed in the CBCT procedure across all dimensions, as well as in the kV procedure along the X and Y axes. Finally, for the “cranial” area, significant differences emerged in the CBCT procedure along the X and Y axes, and in the kV procedure along the Y axis.

While this study demonstrates statistically significant differences supporting the use of asymmetric planning margins in select directions, the actual clinical significance - particularly regarding dose reduction to organs at risk (OARs) or improvement in target coverage - remains to be quantified. Prospective dosimetric studies are needed to assess whether these asymmetric margin adaptations translate to a meaningful reduction in irradiated normal tissue or improved clinical outcomes.

Overall, this study reinforces the clinical importance of tailored image-guided strategies and margin design. Future research should further explore intra-fractional motion management and real-time adaptive imaging techniques [4,6,9,11,15].

There are maybe some limitations in the present study. Inter-observer variability was not taken into account, and this could potentially influence the reproducibility of the results. Additionally, although the sample size was deemed sufficient for statistical analysis, some of the subgroups (e.g., cranial cases) had a rather low number of cases, therefore reducing the statistical power of some of the comparisons. Also, body mass index, which could potentially influence the magnitude of the measurements, was not included in this analysis. Additional limitations include the absence of a formal assessment of image quality as a modifier of setup accuracy and the lack of direct evaluation of the dosimetric or clinical impact resulting from margin adaptations. Moreover, while acquisition protocols were standardized as much as possible, some area-specific variations may reduce overall protocol consistency. Despite these gaps, the core comparative findings are robust. Finally, the study contains data from a single Radiotherapy Department, a fact that might limit the generalizability of the findings. Future multicenter studies with standardized protocols could confirm the clinical applicability of asymmetric margins across different anatomical areas.

## Conclusions

This study provides a detailed comparative evaluation of setup errors between CBCT and planar (kV) imaging techniques across four anatomical areas. CBCT was associated with higher deviations in specific spatial dimensions, particularly along the X-axis in the prostate, lung, and cranial areas. Moreover, MANOVA analysis revealed significant contributions from the anatomical area and treatment day to setup variability, emphasizing the importance of individualized image guidance protocols. No significant differences were identified between morning and afternoon imaging sessions, supporting procedural consistency throughout the treatment day.

The study's analysis of directional deviations identified statistically significant differences between positive and negative setup errors in selected regions, justifying the use of asymmetric van Herk margins, but this important issue may warrant further study. This approach may offer enhanced accuracy and dose conformity in clinical practice, especially for lung and cranial tumors. In conclusion, the findings support the continued use of both CBCT and planar imaging, with CBCT offering volumetric advantages in certain contexts. Moreover, the use of data-driven asymmetric margins represents a promising strategy to optimize treatment precision in RT. While the results of the present dosimetric analysis are promising, immediate implementation of the proposed margin adaptations into routine clinical practice is not recommended without further validation. The data support selective consideration of asymmetric margins in complex cases, but multi-center prospective studies and careful clinical judgment are necessary to ensure patient safety and optimal outcomes.
